# Evaluation of Targeted Alpha Therapy Using [^211^At]FAPI1 in Triple-Negative Breast Cancer Xenograft Models

**DOI:** 10.3390/ijms252111567

**Published:** 2024-10-28

**Authors:** Kaori Abe, Tadashi Watabe, Kazuko Kaneda-Nakashima, Yoshifumi Shirakami, Yuichiro Kadonaga, Sadahiro Naka, Kazuhiro Ooe, Atsushi Toyoshima, Frederik Giesel, Takeshi Usui, Nanae Masunaga, Chieko Mishima, Masami Tsukabe, Tetsuhiro Yoshinami, Yoshiaki Sota, Tomohiro Miyake, Tomonori Tanei, Masafumi Shimoda, Kenzo Shimazu

**Affiliations:** 1 Department of Breast and Endocrine Surgery, Graduate School of Medicine, Osaka University, Suita 565-0871, Japan; abe216@onsurg.med.osaka-u.ac.jp (K.A.);; 2 Department of Radiology, Graduate School of Medicine, Osaka University, Suita 565-0871, Japan; 3 Institute for Radiation Sciences, Osaka University, Suita 565-0871, Japan; 4 Core for Medicine and Science Collaborative Research and Education, Forefront Research Center, Graduate School of Medicine, Osaka University, Suita 560-0043, Japan; 5 Department of Pharmacy, Osaka University Hospital, Suita 565-0871, Japan; 6 Department of Nuclear Medicine, University Hospital Duesseldorf, Medical Faculty, Heinrich-Heine-University, 40225 Duesseldorf, Germany

**Keywords:** triple-negative breast cancer, FAP, CAF, FAPI-PET, Astatine (^211^At), α-emitting nuclides, [^211^At]FAPI1, theranostics

## Abstract

Triple-negative breast cancer (TNBC) presents limited therapeutic options and is associated with poor prognosis. Early detection and the development of novel therapeutic agents are therefore imperative. Fibroblast activation protein (FAP) is a membrane protein expressed on cancer-associated fibroblasts (CAFs) that plays an essential role in TNBC proliferation, migration, and invasion. Consequently, it is hypothesized that the Astatine (^211^At)-labeled FAP inhibitor (FAPI) selectively exerts anti-tumor effects through alpha-particle emission. In this study, we aimed to assess its theranostic capabilities by integrating [^18^F]FAPI-74 PET imaging with targeted alpha therapy using [^211^At]FAPI1 in TNBC models. Mice xenografts were established by transplanting MDA-MB-231 and HT1080 cells (control). As a parallel diagnostic method, [^18^F]FAPI-74 was administered for PET imaging to validate FAP expression. A single dose of [^211^At]FAPI1 (1.04 ± 0.10 MBq) was administered to evaluate the therapeutic efficacy. [^18^F]FAPI-74 exhibited high accumulation in MDA-MB-231 xenografts, and FAP expression was pathologically confirmed via immunostaining. The group that received [^211^At]FAPI1 (n = 11) demonstrated a significantly enhanced anti-tumor effect compared with the control group (n = 7) (*p* = 0.002). In conclusion, [^18^F]FAPI-74 PET imaging was successfully used to diagnose FAP expression, and as [^211^At]FAPI1 showed promising therapeutic efficacy in TNBC models, it is expected to be a viable therapeutic option.

## 1. Introduction

At present, breast cancer is the most prevalent global malignancy [1]. Breast cancer is categorized into four subtypes based on the presence or absence of hormone receptors—estrogen receptor (ER) and progesterone receptor (PgR)—which serve as therapeutic targets, as well as the expression of HER2. Tumors that lack ER, PgR, and HER2 expression are classified as triple-negative breast cancer (TNBC) [2]. TNBC has more limited treatment options than other subtypes. Furthermore, the prognosis for recurrent or stage four breast cancer is notably adverse [3]. Precisely identifying TNBC lesions and developing novel therapeutic agents are crucial objectives.

Cancer-associated fibroblasts (CAFs) are present in malignant tumor tissues, where they create a microenvironment conducive to cancer progression by modulating immune responses and employing various mechanisms. They have been demonstrated to facilitate cancer cell proliferation, angiogenesis, and vascular invasion [4,5]. Fibroblast activation protein (FAP) is a membrane protein expressed on CAFs. It functions as a gelatinase not only in cancerous tissues but also in the fibroblasts and pericytes activated during wound healing [6]. FAP is infrequently expressed in normal tissues but is prevalent in various malignant tumors [7,8]. Recently, FAPI-PET, which targets FAP within the microenvironment surrounding cancer cells, has garnered significant attention. Different from FDG-PET, which measures glucose metabolism in cancer cells [9,10], FAPI-PET has been shown to allow for greater tumor accumulation [9]. Our research findings indicate that FAPI-PET exhibits superior sensitivity compared to FDG-PET in detecting lymph node metastases; it can identify accumulation in smaller lesions that may be challenging to detect with conventional FDG-PET [11].

^211^At (with a half-life of 7.2 h), which emits alpha radiation, is garnering attention as a promising novel nuclear medicine therapeutic agent. It is hypothesized that it exerts significant anti-tumor effects due to its high-energy alpha particles, which transfer energy over a short range and thus minimize damage to the surrounding normal tissues [12]. We previously demonstrated that ^211^At has an outstanding therapeutic effect in preclinical studies using various xenograft models, including thyroid cancer and glioma [13,14]. We hypothesized that conjugating ^211^At with FAPI enhances its anti-tumor efficacy based on our group’s previous research work, in which we successfully labeled ^211^At with FAPI and established a proof of concept for its therapeutic application [15]. If [^211^At]FAPI1 can selectively accumulate at tumor sites expressing FAP, it can exhibit anti-tumor activity against a broad range of tumors, including breast cancer, given that FAP is also present in the triple-negative subtype (TNBC), where it is implicated in proliferation, migration, and invasion [16,17].

As part of a theranostic strategy, we first aimed to verify the accumulation of [^18^F]FAPI-74, a well-established FAPI-PET probe currently employed in clinical settings [18], in various carcinoma models, and confirm FAP expression with immunostaining. Subsequently, we assessed and elucidated the therapeutic impact and efficiency of [^211^At]FAPI1 in a triple-negative breast cancer (TNBC) model.

## 2. Results

### 2.1. [^18^F]FAPI-74 PET/CT Imaging and Biodistribution of Xenografts

**Figure 1:**
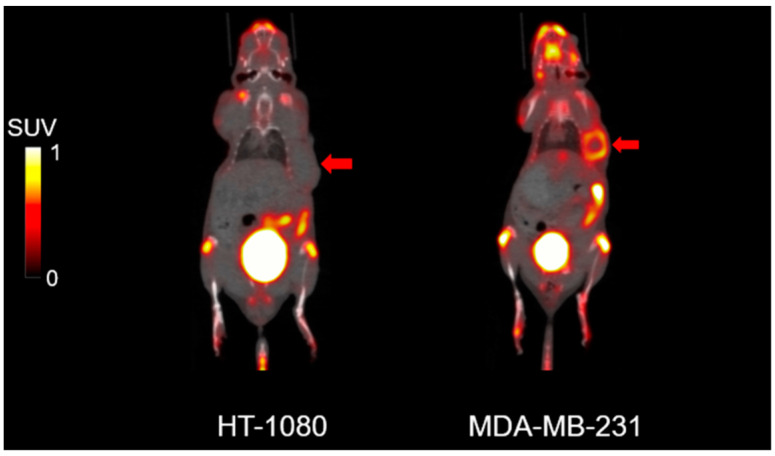
Representative [^18^F]FAPI-74 PET images of HT1080 and MDA-MB-231 xenograft models. The tumors are indicated by arrows.

**Figure 2:**
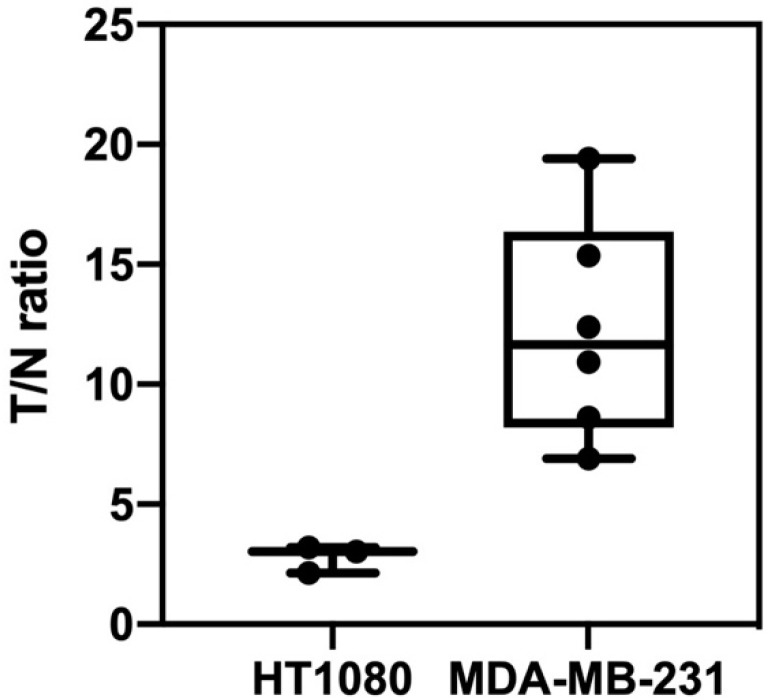
PET-based biodistributions: ratios of tumor SUVmax to normal tissue SUVmean for HT1080 (n = 3) and MDA-MB-231 xenografts (n = 6), with *p* = 0.003.

The PET imaging results are shown in Figure 1. All data from the mice are presented in Supplementary Figures S1 and S2. In comparing MDA-MB-231 (n = 6) and HT1080 xenografts (n = 3), [^18^F]FAPI-74 PET showed higher uptake in the former (median SUVmax, 1.10 [range, 0.97–2.96] with a median T/N ratio of 11.65 [range, 6.90–19.41]) than in the latter (median SUVmax, 0.22 [range, 0.20–0.27] with a mean T/N ratio of 3.03 [range, 2.13–3.20]) (*p* = 0.016, *p* = 0.003, and *p* = 0.016) (Figure 2). No metastatic findings were detected, as verified by PET imaging and autopsy examinations.

### 2.2. Histological and Immunohistochemical Analyses

**Figure 3:**
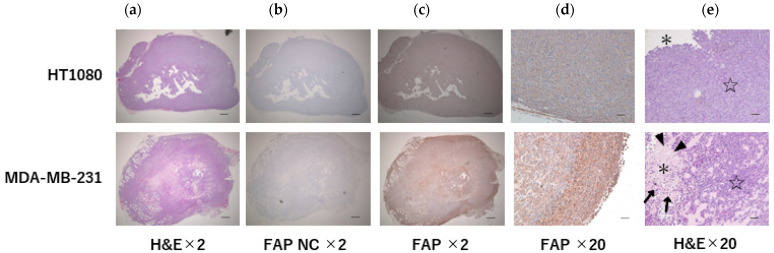
Histological and immunohistochemical staining of xenografts. FAP expression observed in the MDA-MB-231 xenograft. FAP immunohistochemical staining was more intense at the tumor margins. (**a**) Tumor: H&E; (**b**) tumor: FAP immunohistochemical staining negative control; (**c**) tumor: FAP immunohistochemical staining; (**d**) tumor margin: FAP immunohistochemical staining; (**e**) border between the center (asterisk) and the margin (star) of the tumor. In MDA-MB-231, xenograft karyolysis, cytoplasmic swelling (arrow), apoptotic bodies, and chromatin aggregation (arrowhead) were observed; in the central legions: H&E was observed. NC: negative control; ×2 images; scale bars = 500 μm, ×20 images; scale bars = 50 μm.

**Figure 4:**
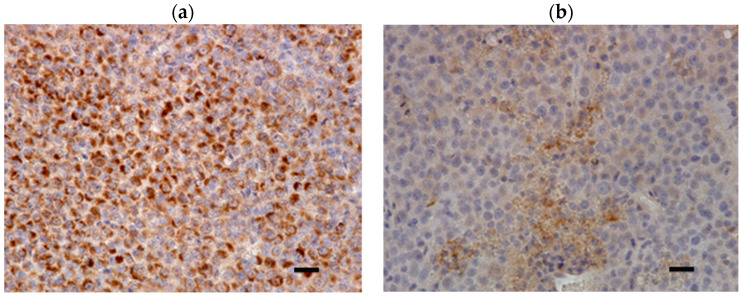
Representative FAP staining of MDA-MB-231 xenograft (high magnification). FAP expression was noted in the (**a**) tumor cells and (**b**) stroma. Scale bars = 25 μm.

Tumor sections from all the mice were subjected to staining with hematoxylin and eosin (H&E) and anti-FAP alpha antibody. In the MDA-MB-231 xenograft, the features of necrosis, such as karyolysis and cytoplasmic swelling, and the features of apoptosis, such as apoptotic bodies and chromatin aggregation, were found in the center of the legion via H&E staining. The immunohistochemical analysis revealed pronounced FAP expression in the MDA-MB-231 xenografts. FAP staining was notably more intense at the tumor margins, aligning with the PET imaging results. In contrast, the cellular appearance within the tumor was uniform in the HT1080 xenografts and somewhat sparse in the center. FAP immunohistochemical staining was weak in the HT1080 xenografts (Figure 3). Both immunohistochemical staining and PET imaging corroborated the presence of FAP expression. In MDA-MB-231 xenografts, FAP expression was noted in both the tumor cells (Figure 4a) and the stroma (Figure 4b).

### 2.3. Biodistribution of [^211^At]FAPI1 in MDA-MB-231 Xenograft

**Figure 5:**
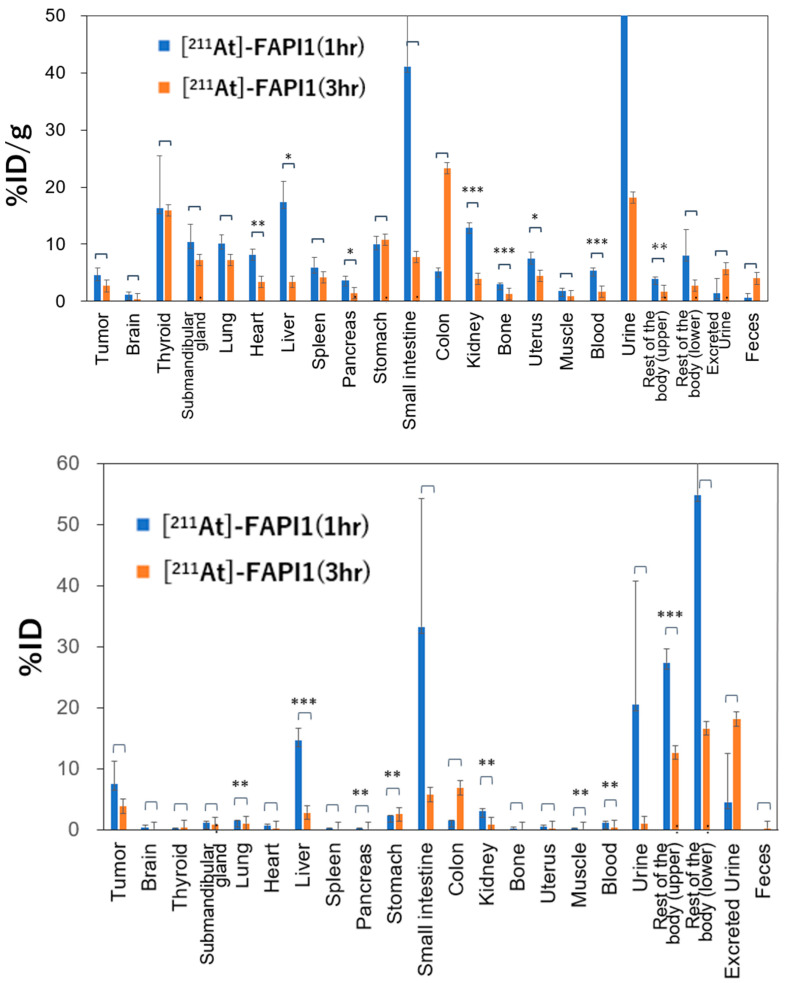
%ID/g and %ID of [^211^At]FAPI1 in various organs at 1 h and 3 h post-injection. * *p* < 0.05, ** *p* < 0.01, and *** *p* < 0.005.

The biodistribution of [^211^At]FAPI1 is illustrated in Figure 5. In most organs, the accumulation of [^211^At]FAPI1 decreased after 3 h, indicating washout. More specifically, the tumor accumulation was 4.48%ID/g at 1 h and 2.70%ID/g at 3 h, reflecting a temporal decline. The observed washout from the liver and kidneys suggests that the hepatic and renal pathways are the primary excretion routes. Over time, accumulation in the small intestine diminished, while it increased in the large intestine, with bile-derived radioactive isotopes (RIs) migrating from the small intestine. Finally, thyroid accumulation was slightly elevated, potentially due to the release of ^211^At.

### 2.4. Changes in Tumor Size and Body Weight

**Figure 6:**
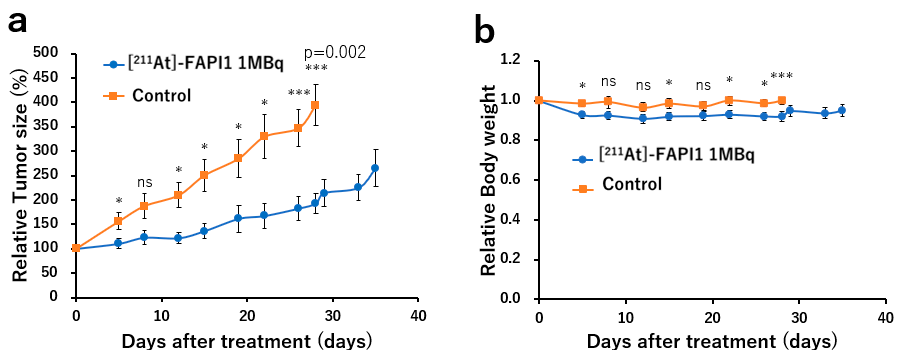
Changes in tumor size and relative body weight in [^211^At]FAPI1 and control groups. (**a**) Relative tumor size and (**b**) relative body weight. * *p* < 0.05 and *** *p* < 0.005; ns = not significant.

The changes in the relative tumor size and body weight in the [^211^At]FAPI1-treated group versus the control group are depicted in Figure 6.

The relative tumor size was significantly smaller in the [^211^At]FAPI1-treated group than in the control group, with substantial differences being observed from day 12 onwards and becoming more pronounced over time (*p* = 0.002) (Figure 6a). A slight reduction in body weight was noted in the [^211^At]FAPI1-treated group relative to the control group, with the decrease being limited to 8% in the former group (Figure 6b). The absolute tumor size change is presented in Supplementary Figure S3.

## 3. Discussion

In this study, we demonstrated that [^211^At]FAPI1 exhibited therapeutic efficacy in a TNBC model. We also proved the utility of [^18^F]FAPI-74 in PET imaging, with immunostaining confirming FAP expression in MDA-MB-231 tumor cells. FAP was found to be expressed not only in the surrounding microenvironment but also in the TNBC itself.

[^18^F]FAPI-74 exhibited tumor accumulation in the TNBC model. This model has proven effective for experimental systems involving FAP with cell line-derived xenografts (CDXs). MDA-MB-231 was chosen as the TNBC cell line in this study for two key reasons: it is the most commonly utilized cell line [19] and although MDA-MB-231 does not inherently express FAP, it does so when co-cultured with fibroblasts in tumor tissues. The interaction between MDA-MB-231 and CAFs is reported to enhance tumor growth and elevate invasive potential [20].

In this study, [^18^F]FAPI-74 demonstrated particularly notable accumulation at the tumor margins. While low accumulation within the tumor was initially attributed to necrosis, the histopathological analysis indicated that the latter was not extensive. Thus, the model suggests a significant propensity for FAP expression at the tumor margins. Additionally, a high number of tumor-infiltrating lymphocytes (TILs) was observed via H&E staining, which may imply an association between FAP expression and tumor immunity. Although it is commonly accepted that fibrosis is less prevalent and FAP production is lower in CDX models [21], our study’s findings confirm [^18^F]FAPI-74 accumulation. Tumor PET accumulation was significantly greater in the MDA-MB-231 than in the HT1080 xenografts, where the latter were used as an FAP negative control [22]. Given that CDX models are more practical than patient-derived xenografts (PDXs) or transgenic mice, these findings are valuable for CDX-based TNBC models.

We are considering a strategy to accumulate [^211^At]FAPI1 in FAP-expressing CAFs, which will then emit alpha rays to target tumors. Clinically, FAP expression is predominantly observed in CAFs. However, in our study using MDA-MB-231 xenografts, FAP expression was observed in both the tumor cells and the stroma. Since this treatment targets FAP expressed in CAFs, it would be ideal to use a tumor-bearing model where these are consistently present. However, developing such a model remains challenging.

In this study, because we aimed to investigate FAPI-targeted therapy, only FAP staining was performed. In breast cancer, CAF markers such as FAP, α-SMA, Vimentin, FSP1, PDGFRα, PDGFRβ, Caveolin-1, and PDPN have been validated [23]. These markers, including those of apoptosis, should be investigated in future studies.

FAP-targeted cancer therapy has garnered significant attention in recent years. Cancer growth is not solely driven by malignant cells, as it also involves non-autonomous mechanisms that are mediated by the surrounding microenvironment. Cancer cells interact with and alter normal cells in adjacent tissues, circumventing growth-suppressive signals and actively modifying the environment throughout their development and evolution [24]. The cancer microenvironment also plays a crucial role in therapeutic resistance, exerting immunosuppressive effects. Recent advancements include immune checkpoint inhibitor (ICI) therapies aimed at neutralizing these suppressive signals [24]. The results of several studies indicate that the combination of FAPI-targeted therapy with immune checkpoint inhibitors (ICIs) enhances anti-tumor effects [25,26,27,28]. Drug delivery systems (DDSs) are critical for maximizing anti-tumor efficacy and minimizing side effects. An effective DDS ensures that a drug (1) targets the intended organ, (2) permeates the organ’s tissues, (3) reaches the specific target cells, and (4) interacts with the desired organelles and molecules within the cells, thereby achieving its therapeutic effect. Generally, fibrosis within the cancer microenvironment impedes drug delivery due to the reduced blood flow in fibrotic tissues. As a DDS formulation, FAPI-targeted therapy holds promise for enhancing the anti-tumor effects of conventional therapies by disrupting the cancer microenvironment, including fibrotic structures.

On the other hand, as an alpha-emitting nuclide with a short range, ^211^At is hypothesized to selectively induce anti-tumor effects while having minimal effect on adjacent normal cells [29,30]. Compared to the conventionally used beta ray-emitting radionuclides for cancer treatment, alpha ray-emitting radionuclide-based therapy has a significantly higher probability of causing double-strand breaks and is more cytotoxic [31]. With our study as an example, the quantity of the compound utilized in targeted alpha therapy is less than a microdose and does not exhibit any pharmacological activity. [^18^F]FAPI-74 accumulation in the MDA-MB-231 xenografts suggests that [^211^At]FAPI1 was effectively taken up by cancer-associated fibroblasts (CAFs) and exerted anti-tumor effects. ^211^At is particularly suited for FAPI-targeted therapy, as its relatively short half-life of 7.2 h enables higher radioactivity to be delivered to the tumor early on post-administration, thereby increasing the locally absorbed dose [12]. Upon comparing the distribution of [^211^At]FAPI1 at 1 and 3 h post-administration, we found a general washout trend, though variations among the organs were noted. The increased accumulation observed in the colon may have been due to intestinal excretion, while the elevated levels of [^211^At]FAPI1 in the thyroid and stomach at 3 h were likely due to dehalogenation.

In order to enhance tumor accumulation, according to recent reports, we propose two strategies. Using an albumin binder improves blood retention, facilitating continuous accumulation in tumors [32]. This makes it valuable for both diagnostic imaging and anti-tumor effects. Another approach is to form dimers. Homodimeric FAP inhibitors have been reported to exhibit high FAP affinity, significant tumor uptake, prolonged tumor retention, and reduced accumulation in other organs, making them valuable for both diagnosis and therapy [33]. Of the currently available alpha-emitting radionuclides, only a few have characteristics suitable for clinical application [31]. In addition to their short half-life, the difficulty in obtaining them has been an obstacle to the development of targeted alpha therapy. Future developments are anticipated.

Treatment options for TNBC are being explored daily. In recent years, ICIs, which act in the peritumor microenvironment to regulate tumor immunity, have become available for preoperative chemotherapy, improving the outcomes of TNBC [34]. CAFs are present in the tumor microenvironment and have been reported to be involved in immune escape in TNBC [35]. Therapeutic approaches to the tumor microenvironment may be very effective in treating this type of breast cancer. [^211^At]FAPI1 administration selectively incorporates ^211^At into FAP expression sites, with the α rays not only directly exerting their anti-tumor effect but also being incorporated into the CAFs. Based on the regulation of the latter, we expect that the peritumor microenvironment can also be regulated and a synergistic effect may be obtained. For refractory metastatic recurrent TNBC expressing FAP, [^211^At]FAPI1 administration is expected to have a therapeutic effect on the metastatic site, including small lesions.

This study had several limitations. Firstly, this research work was constrained by the small number of mice due to institutional restrictions on the use of alpha-emitting radioisotopes, as dictated by legal regulations. Future studies should involve a larger sample size and include appropriate controls for a more comprehensive evaluation over extended observation periods. Since there are no previous studies using similar models for the [^211^At]FAPI1 compound, we will increase the number of samples in future experiments. Given that we utilized the most commonly used cell lines, we expect that our results will be referenced in subsequent studies. Secondly, although the amount of the compound used for alpha radiation therapy does not demonstrate pharmacological activity, its effect cannot be completely ruled out. As a control, it may be prudent to include a FAPI1 group without astatine labeling. Future studies will thus consider dose-dependent studies. In addition, [^211^At]FAPI1 exhibited suboptimal distribution within tumors compared to other organs, indicating a need to increase accumulation. Once a compound with improved tumor retention is developed, further research should establish optimal dosing regimens and assess toxicity. Additionally, as the tumors did not fully regress after a single administration and exhibited regrowth over time, multiple doses are likely required. Subsequent studies should aim to confirm the compound’s life-prolonging effects in tumor-bearing mice before advancing to human clinical trials. If preclinical safety is established, clinical application in humans should be considered. Furthermore, we did not stain the markers of CAFs. In the xenografts, FAP expression was observed in the tumor cells as well as in what appeared to be the stroma. In humans, a more robust stroma and cancer-associated fibroblasts are formed, making it challenging to construct a more suitable tumor-bearing model for future evaluations, including assessments with other CAF markers. Finally, this study was limited to MDA-MB-231 and HT1080 tumor models. Future research should explore other TNBC strains, as well as different subtypes (such as ER- or HER2-positive cancers) and other carcinomas. Although we believe that the therapeutic effect observed in this study is due to DNA double-strand breaks caused by alpha radiation, in the future, we would like to further explore background factors such as radio resistance-associated genes, as well as evaluate immune effects through single-cell sequencing analysis [36].

## 4. Materials and Methods

### 4.1. Preparation of Xenograft Models

The animals were prepared according to a previously published protocol [37]. MDA-MB-231 (human triple-negative breast cancer cells) and HT-1080 cells (human fibrosarcoma cells) were sourced from the American Type Culture Collection. The MDA-MB-231 cells were cultured in Dulbecco’s Modified Eagle Medium (DMEM)/Ham’s F-12 medium supplemented with 10% fetal bovine serum (Sigma-Aldrich, St. Louis, MO, USA) at 37 °C in a humidified incubator with 5% CO_2_, while the HT-1080 cells were maintained in RPMI 1640 medium with L-glutamine and phenol red (FUJIFILM Wako Pure Chemical, Osaka, Japan), supplemented with 10% heat-inactivated fetal bovine serum and 1% penicillin–streptomycin. The cells were washed with phosphate-buffered saline (PBS) and harvested with trypsin. Female NOD/ShiJic-scid Jcl mice and male BALB/c Slc-nu/nu mice were procured from CLEA Japan Inc. (Tokyo, Japan) and SLC Japan (Shizuoka, Japan), respectively. Tumor xenograft models were established by injecting 1–10 × 10^6^ cells of MDA-MB-231 or HT-1080, suspended in 0.1 mL of culture medium and Matrigel^®^ (1:1; Corning Inc., Corning, NY, USA), into the mammary fat pad of NOD/ShiJic-scid Jcl mice or nude mice. For PET-CT imaging, the tumors had to range from 6 to 16 mm in diameter and be in the 1-to-5-week growth phase before [^18^F]FAPI-74 administration. To evaluate the therapeutic effect of ^211^At, the tumors had to measure approximately 14 mm in diameter and 1000 mm^3^ in volume and be in the 4-to-5-week growth phase before [^211^At]FAPI1 administration.

The study protocol was approved by the Osaka University Graduate School of Science Animal Care and Use Committee (approval number 04-070-006) and was in accordance with Osaka University Animal Experimentation Regulations.

### 4.2. [^18^F]FAPI-74 Synthesis

[^18^F]FAPI-74 solution was synthesized using CFN-MPS200 (Sumitomo Heavy Industries, Tokyo, Japan) according to a previously published method [38]. [^18^F] fluoride eluted with 0.5 M sodium acetate buffer and precursor solution was mixed and fluorinated for 5 min at room temperature, followed by 15 min at 95 °C. [^18^F]FAPI-74 was purified using an HLB cartridge and ethanol. Finally, the solution was obtained by diluting [^18^F]FAPI-74 with 10 mM phosphate-buffered saline containing 100 mg of sodium ascorbate, followed by filtering. The radiochemical purity was greater than 95%. Further detailed information is provided in Supplementary Information S4.

### 4.3. PET/CT Scanning

PET/CT images were obtained using a small-animal PET scanner (Siemens Inveon PET-CT, Dallas, TX, USA). The MDA-MB-231 xenograft mice (9–10 weeks old; body weight = 18.2 ± 3.2 g; n = 6) were imaged 4 weeks post-implantation and [^18^F]FAPI-74 (10.6 ± 2.2 MBq) was administered via the tail vein under 2% isoflurane anesthesia. Similarly, HT1080 xenograft mice (9 weeks old; body weight = 20.3 ± 2.6 g; n = 3) were also imaged under 2% isoflurane anesthesia and were injected [^18^F]FAPI-74 (10.56 ± 1.1 MBq) via the tail vein.

Static PET scans (scan duration = 10 min) were performed 1 h after injection, followed by a CT scan. PET data were reconstructed into a single frame using three-dimensional ordered-subset expectation–maximization (16 subsets and 2 iterations), with attenuation and scatter correction applied. Regions of interest were drawn on the muscles, heart, lungs, liver, gallbladder, kidneys, intestine, and tumor. Standardized uptake values (SUVs) were calculated using AMIDE software (version 1.0.4). SUV is a semi-quantitative index adjusted for individual dosage and body weight. Additionally, the tumor to normal tissue ratio (T/N ratio) of SUV within the same individual was calculated by setting the region of interest (ROI) on the thigh muscle opposite the tumor as the reference normal tissue.

### 4.4. [^211^At]FAPI1 Synthesis

[^211^At]FAPI1 was prepared as described in a previous paper [15]. In brief, ^211^At was obtained from RIKEN (Wako, Saitama, Japan) and the Research Center for Nuclear Physics at University of Osaka through a supply platform for short-lived radioisotopes. ^211^At was dissolved in pure water and reacted with a FAPI precursor molecule coupled with a dihydroxyboryl group in the presence of potassium iodide as a catalyst in a weak basic aqueous solution at 80 °C for 45 min. Both the radiochemical yield and purity of ^[211^At]FAPI1 were greater than 97.0%. Further detailed information is provided in Supplementary Information S5.

### 4.5. Biodistribution of [^211^At]FAPI1

MDA-MB-231 xenograft mice (body weight = 22.1 ± 0.5 g) were utilized to assess biodistribution following [^211^At]FAPI1 administration (n = 6: 0.1–1.2 MBq). After euthanasia under deep anesthesia induced by isoflurane inhalation at 1 (n = 3) and 3 h (n = 3) post-administration, the thyroid gland, salivary glands, heart, lungs, stomach, stomach contents, small and large intestines, pancreas, liver, spleen, kidneys, testis, urine, blood, and tumor were collected and weighed for biodistribution analyses. Radioactivity was measured using a gamma counter (AccuFLEX γ7000; Aloka, Tokyo, Japan) with cross-calibration. The results are expressed as %ID and %ID/g.

### 4.6. [^211^At]FAPI1 Therapy

MDA-MB-231 xenograft mice were placed into two groups: (1) a treatment group administering [^211^At]FAPI1 (n = 11; body weight = 20.4 ± 0.7 g; tumor volume = 1062 ± 327 mm^3^) and (2) a control group receiving no treatment (n = 11; body weight = 21.0 ± 0.4 g; tumor volume = 1090 ± 169 mm^3^). The mice in the treatment group were intravenously injected with approximately 1.04 ± 0.10 MBq of [^211^At]FAPI1 (0.3 µg per subject) via the tail vein in a single dose. The tumor size was measured twice a week using digital calipers, and the tumor volume was calculated using the following formula: volume (mm^3^) = 4/3 × π × length/2 (mm) × width/2 (mm)^2^.

### 4.7. Histology and Immunohistochemistry

All the mice were euthanized after [^18^F]FAPI-74 PET imaging, and the tumor xenografts were excised. Immunohistochemical staining was conducted using an anti-FAP alpha antibody (ab53066; Abcam, Cambridge, UK) and the Dako EnVision + System HRP-Labelled Polymer Anti-Rabbit (K4003) (DAKO Corp., Glostrup, Denmark). The [^211^At]FAPI1-treated mice were euthanized when the tumor diameter exceeded 20 mm or upon the appearance of ulceration, necrosis, infection, gait disturbance, or alterations in water and food intake. The tissues were fixed in 10% neutral buffered formalin for paraffin embedding and subsequently stained with hematoxylin and eosin (H&E). Tumor blocks from all the mice were also subjected to staining.

### 4.8. Statistical Analysis

The results are expressed as means ± standard deviations. Comparisons between the two groups were conducted using an unpaired *t*-test in Microsoft Excel (version 2019). Differences were considered statistically significant at *p* < 0.05.

## 5. Conclusions

In this study, we demonstrated the feasibility of a theranostic approach for TNBC. [^18^F]FAPI-74 PET revealed substantial accumulation in the TNBC model, correlating with the FAP expression ascertained via immunohistochemistry. [^211^At]FAPI1 exhibited a therapeutic effect in the TNBC model without notable toxicity, indicating that it holds promise as a therapeutic agent; thus, further evaluations, along with greater tumor retention, are necessary.

## Data Availability

Data are available upon request.

## Supplementary Materials

The following supporting information can be downloaded at https://www.mdpi.com/article/10.3390/ijms252111567/s1.
