# Supplementary material for: Evaluation of Targeted Alpha Therapy Using [211At]FAPI1 in Triple-Negative Breast Cancer Xenograft Models
Source: Int J Mol Sci. 2024 Oct 28;25(21):11567. doi: 10.3390/ijms252111567 (PMC11547022; doi:10.3390/ijms252111567)
Supplement: Supplementary file 1 [file ijms-25-11567-s001.zip › ijms-3234615-supplementary/Supplementary Info Figures S1-S3.pdf]

Supplementary Figure S1 FAPI-PET PET images; MDA-MB-231

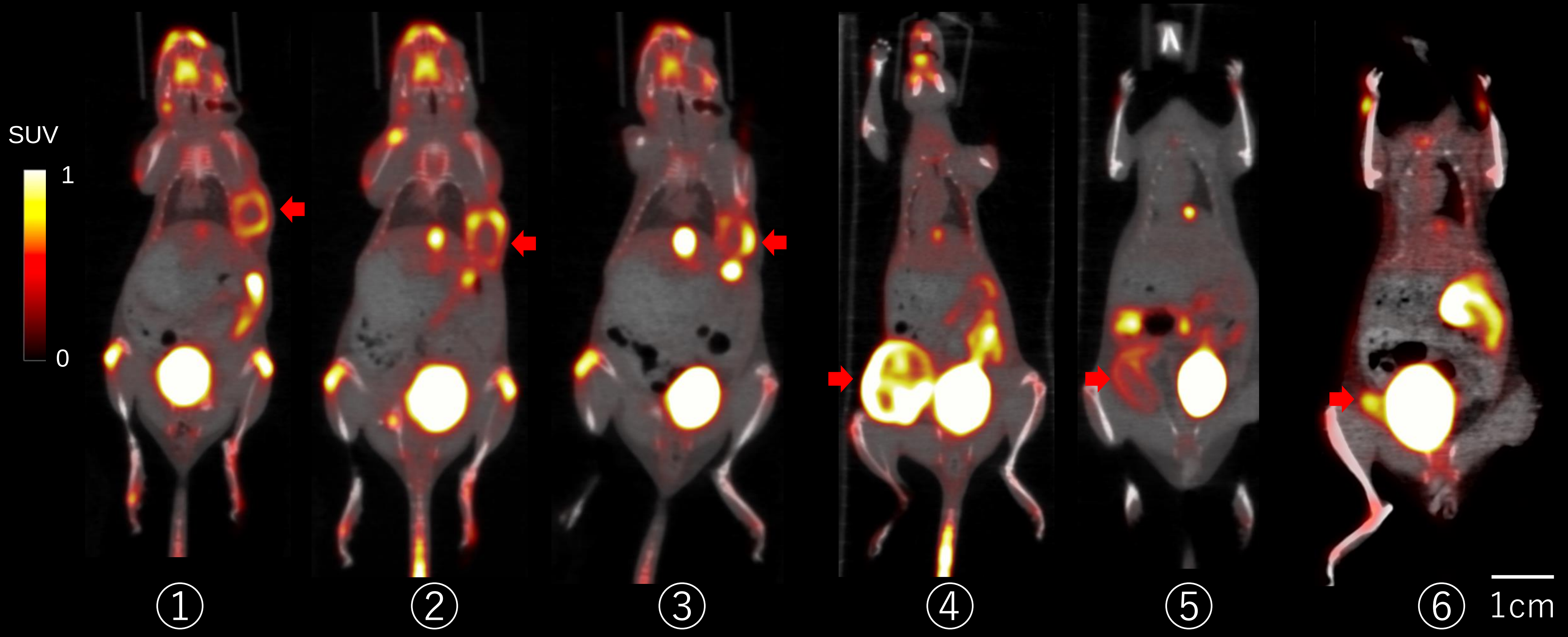

Supplementary Figure S1: [18F] FAPI-74 PET images; MDA-MB-231 xenograft model. The tumors are indicated by arrows. Tumor size ①9.0 x 8.8mm, ②9.1 x 6.4mm, ③7.9 x 6.2mm, ④15.9 x 13.2mm, ⑤11.8 x 6.8mm, ⑥5.6 x 4.3mm

Supplementary Figure S2 FAPI-PET PET images; HT1080

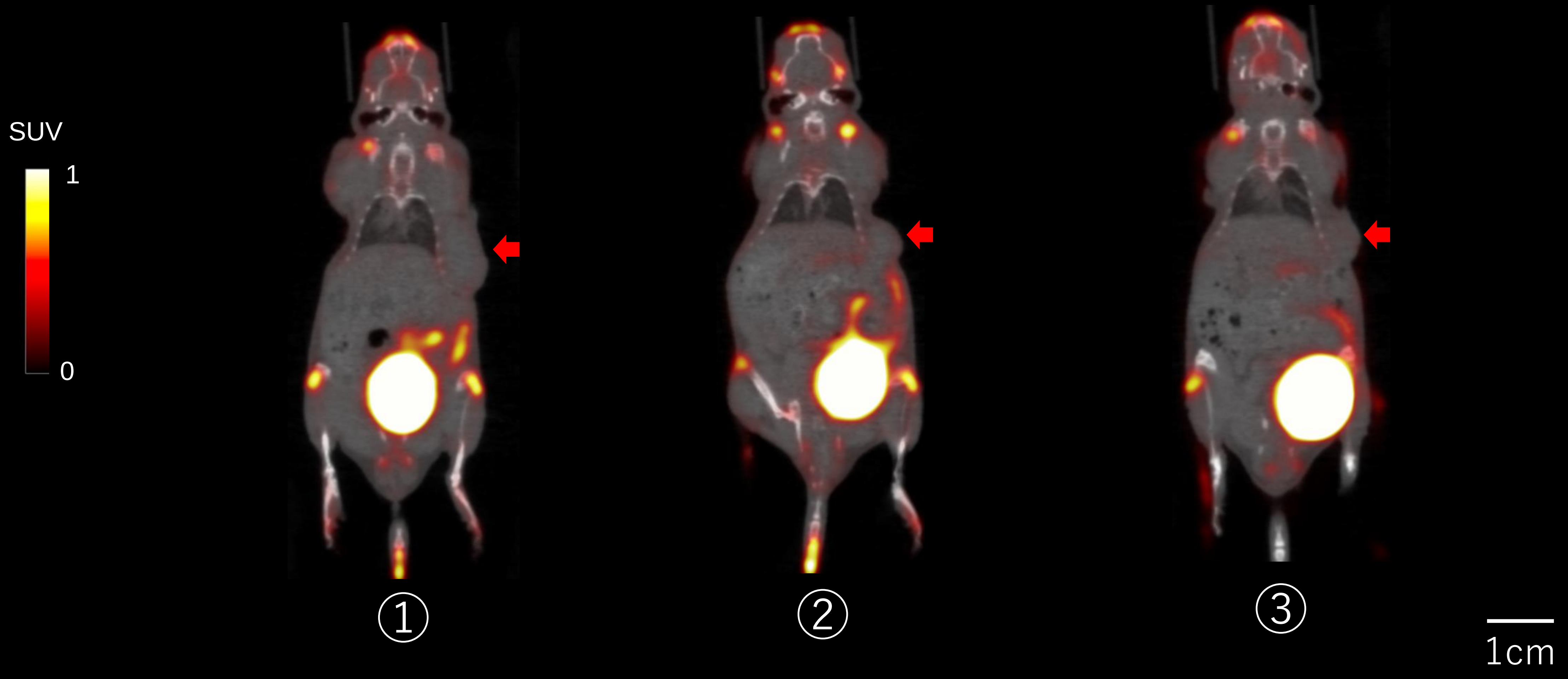

Supplementary Figure S2: [18F] FAPI-74 PET images; HT1080 xenograft model. The tumors are indicated by arrows. Tumor size ①10.8 x 10.1mm, ②8.5 x 7.8mm, ③9.2 x 7.3mm

Supplementary Figure S3 [^{211}At] FAPI1 Tumor size

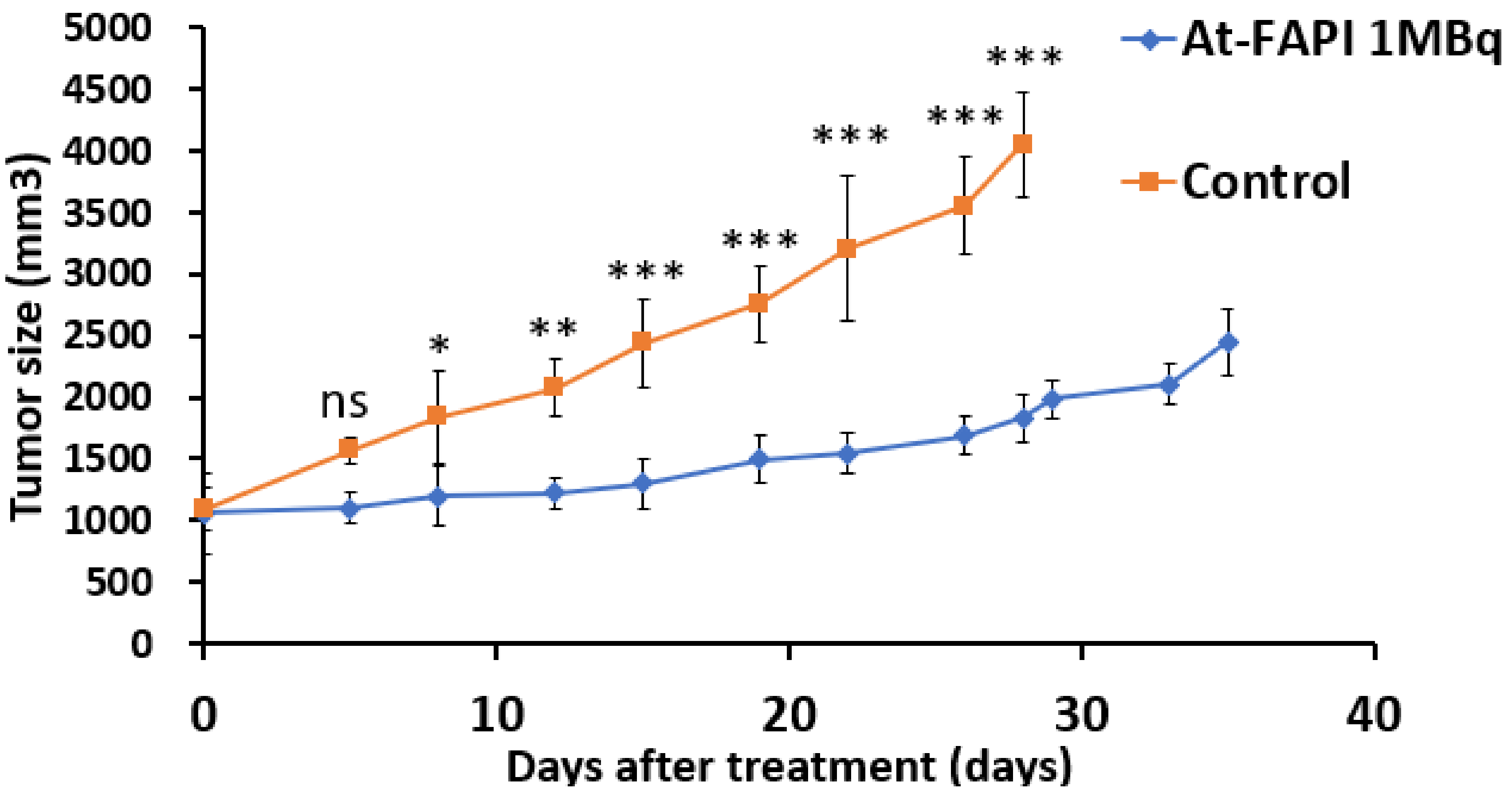

Supplementary Figure S3: Changes in the tumor size in [211At] FAPI1 and control group. * $p<0.05$, ** $p<0.01$, *** $p<0.005$, ns=not significant; t-test
