# Supplementary material for: Evaluation of Targeted Alpha Therapy Using [211At]FAPI1 in Triple-Negative Breast Cancer Xenograft Models
Source: Int J Mol Sci. 2024 Oct 28;25(21):11567. doi: 10.3390/ijms252111567 (PMC11547022; doi:10.3390/ijms252111567)

Supplementary Information S4

Preparation of [^{211}At]FAPI1

Astatine-211 (^{211}At), which was produced by a cyclotron using a nuclear reaction of ^{209}Bi(He, 2n)^{211}At, was purchased from the National Institute for Quantum Science and Technology (QST, Takasaki, Gunma, Japan) and RIKEN (Wako, Saitama, Japan) through a supply platform for short-lived radioisotopes. The obtained ^{211}At was purified by a dry distillation method, and dissolved in pure water. FAPI1, (S)-(4-(2-((2-(2-((4-((2-(2-cyanopyrrolidin-1-yl)-2-oxoethyl)carbamoyl)quinolin-6-yl)oxy)ethoxy)ethyl)amino)-2-oxoethyl)phenyl)boronic acid, was prepared via chemical synthesis according to previously published schemes [17]. In a polypropylene tube, 5 µL of FAPI1 (1 mg/mL aqueous solution), 90 µL of pure water, 30 µL of potassium iodide solution (0.1 mole/L), 10µL of sodium hydrogen carbonate solution (7%) and 20 MBq of ^{211}At aqueous solution were added, and the mixture was reacted at 80 ºC for 45 minutes. The radiochemical purity (RCP) of [^{211}At]FAPI1 were analyzed by TLC (Silica gel G60/F254, Merck, Acetonitrile/Water(2/1)). RCP:>97%. The molecular structures of FAP1 and [^{211}At]FAPI1, and the reaction scheme are shown bellow.

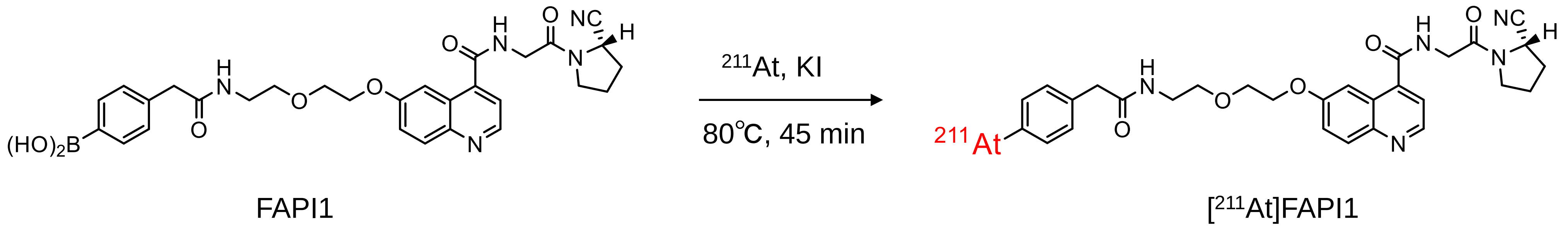

Supplement: Supplementary file 1 [file ijms-25-11567-s001.zip › ijms-3234615-supplementary/Supplementary Info S4 Preparation of [211At]FAPI1.pdf]
