# Supplementary material for: Evaluation of Targeted Alpha Therapy Using [211At]FAPI1 in Triple-Negative Breast Cancer Xenograft Models
Source: Int J Mol Sci. 2024 Oct 28;25(21):11567. doi: 10.3390/ijms252111567 (PMC11547022; doi:10.3390/ijms252111567)
Supplement: Supplementary file 1 [file ijms-25-11567-s001.zip › ijms-3234615-supplementary/Supplementary Info S5 Preparation of [18F]FAPI-74.pdf]

Supplementary Information S5

Preparation of [^{18}F]FAPI-74

[^{18}F]FAPI-74 solution was synthesized by using CFN-MPS200 (Sumitomo Heavy Industries) according to a previously published method [33]. [^{18}F]fluoride eluted with 300 µL of 0.5 M sodium acetate buffer (pH 3.95 ± 0.05) and precursor solution (300 µL of dimethyl sulfoxide, 6 µL of 10 mM aluminum chloride, 4 µL of 20% w/v ascorbic acid, and 20 µL of 4 mM FAPI-74 precursor) was mixed and fluorinated for 5 min at room temperature, followed by 15 min at 95°C. After fluorination, [^{18}F]FAPI-74 was trapped by passing the reaction solution diluted with 10 mL of saline through an HLB cartridge. After washing the cartridge with 3 mL of saline, [^{18}F]FAPI-74 was recovered with 1 mL of ethanol. Finally, the solution was obtained by diluting [^{18}F]FAPI-74 with 14 mL of 10 mM phosphate-buffered saline containing 100 mg of sodium ascorbate, followed by filtering with Millex GV. The radiochemical purity was greater than 95 %.The molecular structures of FAP-74 precursor and [^{18}F]FAPI-74, and the reaction scheme are shown bellow.

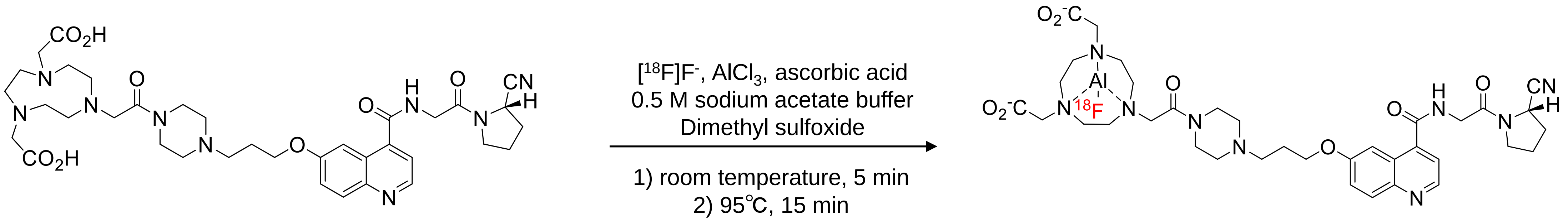

FAPI-74 precursor

[^{18}F]FAPI-74
